# Incidental finding of a bilateral absence of the superior vena cava

**DOI:** 10.1002/ccr3.7224

**Published:** 2023-04-22

**Authors:** Yosef Al‐Sewaidi, Robert Smaczny, Alexander Ranft

**Affiliations:** ^1^ Department for Interventional Radiology and Neuroradiology Klinikum Hochsauerland Arnsberg Germany

**Keywords:** computed tomography, congenital abnormalities, superior vena cava, venous anomaly

## Abstract

Bilateral absence of the superior vena cava is associated with rhythm and structural abnormalities and is diagnosed incidentally either during imaging procedures or venous catheterization or pacemaker implantation. Knowledge of this entity is important to allow proper referral, medical management of its associated abnormalities, and risk reduction in certain interventions.

## Graphical abstract

Venous circulatory anomalies are very rare and usually diagnosed incidentally or during complicated cardiac interventions. Knowledge of them is crucial so as to reduce complication rate for interventions and to allow proper referral for the investigation of possibly associated abnormalities.

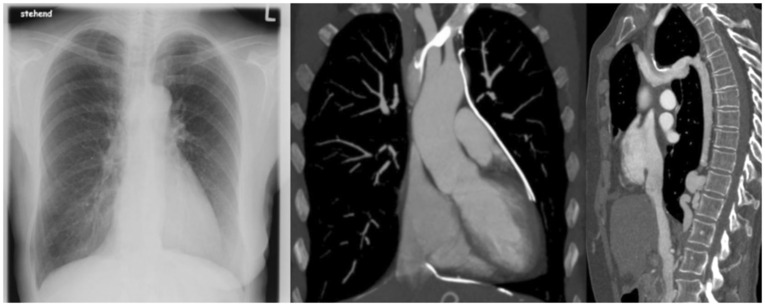

## 1 INTRODUCTION

Variations of the venous circulation can either affect the superior or the inferior vena cava and have highly variable symptomatology with diagnosis being mostly incidental. In our case we report the incidental finding and workup of a bilateral absence of the superior vena cava in a patient without suggestive symptoms.

Systemic venous anomalies afflicting the superior vena cava (SVC) are rare developmental anomalies with origins during embryonic development. Since most patients are asymptomatic,^1^ diagnosis is usually incidental in modalities such as echocardiography, computed tomography (CT), magnetic resonance imaging (MRI), or during unsuccessful cardiac or venous catheterization.^2^ There is a male preponderance, and they can be associated with congenital heart defects, rhythm disturbances, or both.^1^, ^3^, ^4^ Among the SVC anomalies the most frequent ones are a presence of bilateral SVC and an absence of right SVC with a persistent left SVC^2^, ^5^ however a bilateral absence of the SVC is extremely rare.

## 2 CASE REPORT

A 66‐year‐old female patient presented with a week history of upper abdominal pain. Physical examination as well as laboratory workup were inconclusive. Medical and surgical histories were unremarkable, especially cardiac rhythmic and structural, as well as congenital pathology.

Initial routine chest radiography was unspecific, showing a slightly emphysematous lung without any additional findings, particularly the mediastinal or cardiac silhouette. Working on the suspicion of an acute upper abdominal process, the accompanying physician requested subsequent evaluation by CT of the chest and abdomen. Neither an acute nor a malignant process was found as an underlying cause for the patient's abdominal symptoms.

**FIGURE 1:**
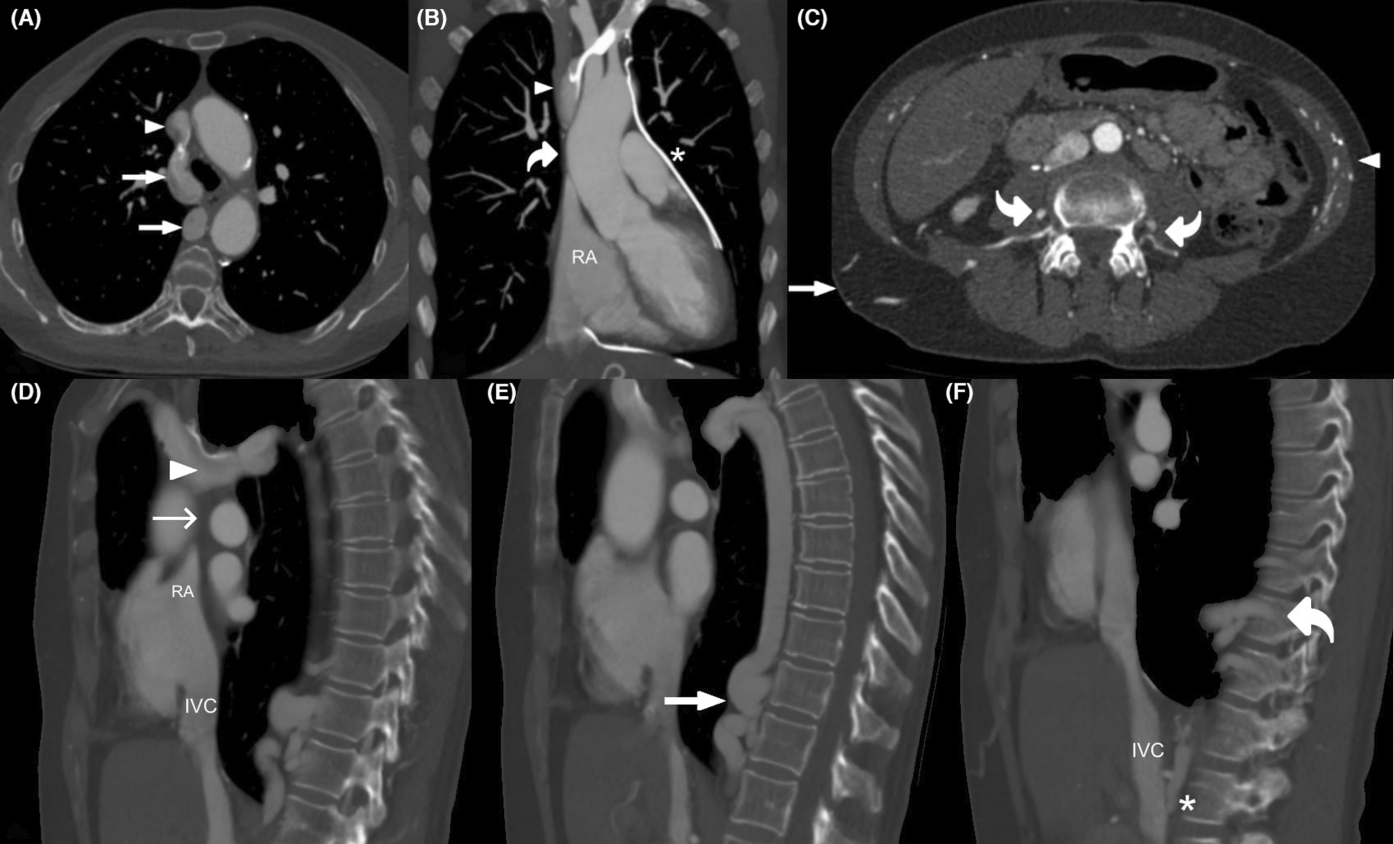
Representation of the main venous drainage pathway of the upper limbs through the azygos and hemiazygos system, as well as its thoracic and abdominal venous collaterals. (A) Junction point of both brachiocephalic veins (arrowhead) draining into the azygos vein (straight arrow). (B) No vascular structure is seen in the mediastinal notch where the SVC should be (curved arrow), leaving the right atrium (RA) with a blind end cranially, which can also be seen in Panel D (thin straight arrow). A pericardiophrenic vein (asterisk) can be seen filled with contrast agent coursing caudally around the heart. (C) Abdominal collaterals in otherwise atypical locations. (D–F) Sagittal reconstruction of the azygos vein in its path with the brachiocephalic vein junction point (arrowhead) draining into the azygos vein (thick straight arrow), eventually joining the inferior vena cava (IVC) distally (asterisk). Additional collaterals in the form of dilated paravertebral/infracostal veins can be seen (curved arrow). The blood is then fed through the IVC back to the right atrium (RA).

Incidentally, a bilateral absence of the SVC in the setting of bilaterally present brachiocephalic veins was noted (Figure 1B,D). These veins joined at the height of the fifth vertebra and drained the upper limbs and head mainly through a common trunk into a dilated varicose‐like azygos and hemiazygos vein that coursed caudally and, fused with the inferior vena cava (IVC) at renal height and the left renal vein (Figure 1 A,B and D–F). Additional dilated and varicose venous collaterals could be observed infracostally (Figure 1F), pericardiophrenically (Figure 1B), as well as within the lateral abdominal musculature and paravertebrally (Figure 1C).

Since the patient had no relevant medical or surgical history, differential diagnosis such as thrombosis or compression of the SVC due to malignancy as well as surgical manipulation of the SVC were excluded.

Having no associated symptoms, such as congestion, headaches, or dyspnea, as well as being hemodynamically stable, the patient was discharged after a short stay on laxatives and proton‐pump inhibitors with the recommendation of evaluation by a cardiologist, in which the patient was to undergo ECG examination, Holter monitoring, and a transthoracic ultrasound for the exclusion of cardiac structural and rhythm abnormalities.

## 3 DISCUSSION

The variations in the venous system can affect both the SVC and IVC, more often the latter^6^, ^7^ and pathogenesis lies at the embryological level.

The development of the systemic venous circulation starts early in the fourth week of embryonic development, as described by Minniti et al.^6^ Involved in it are processes such as the building of anastomoses—bridging—as well as its involutions—regressions.

In an otherwise healthy individual, circulatory development starts with the bilateral anterior cardinal veins (each with a cranial and common part) and the bilateral posterior cardinal veins (each with a caudal and common part). The SVC is formed from the right anterior cardinal vein and the left cranial anterior cardinal vein. The azygos system is formed from the supracardinal veins which arise from the sixth week of gestation. The IVC is formed by the posterior cardinal veins, the subcardinal veins, which arise from the seventh week of gestation, as well part of the supracardinal veins.^6^

Errors in bridging and regressions lead therefore to the myriad of known caval anomalies. Non‐regression of both common anterior cardinal veins leads to a bilateral SVC, whereas regression of the right common anterior cardinal vein and non‐regression of the left common anterior cardinal vein leads to a persistent left SVC.^8^

**FIGURE 2:**
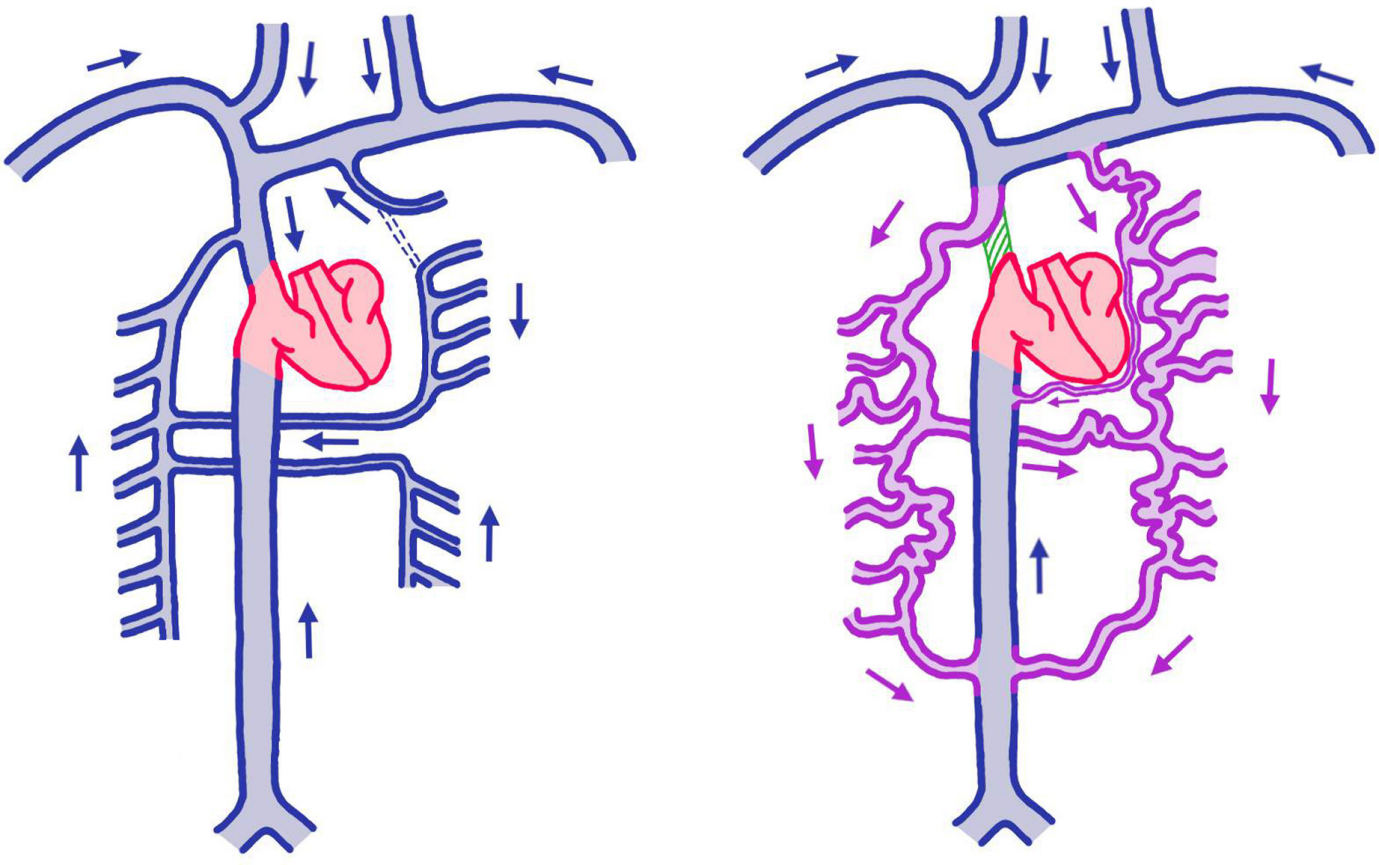
Schematic representation of normal venous circulation (left side) and our patient's circulatory variant (right side). *Left side*: Blood from the lower limbs is fed into the right atrium directly through the IVC. Blood from the upper limbs/head and neck, as well as from the intercostal and lumbar system through the azygos/hemiazygos system is pooled together and fed directly into the right atrium through the SVC; *Right side*: In our patient's case, upper limbs blood, head and neck blood, as well as intercostal and lumbar blood is fed in its entirety through the azygos and hemiazygos system, as well as a pericardiophrenic vein into the IVC and collectively fed into the right atrium through the IVC.

In the case of a bilateral absence of both SVC, this is most likely due to regression of both common anterior cardinal veins. As an adaptive measure, likely analogous to SVC syndrome,^9^ other venous systems tend to dilate and, due to the lack of a rigid wall, become varicose, allowing them to cope with and thus drain the blood from the upper extremities and head, ranging from just the more central azygos system to the more distal pelvic and subcutaneous veins, such as was seen in our case (Figure 2).

Among the superior caval variants, the most frequent ones are the bilateral SVC, as well as a persistent left SVC with an absent right SVC. The case of a bilateral absence of an SVC is extremely rare, having been reported, to our knowledge, 17 times until now (Table 1).

**TABLE 1:** Listing of reported cases of a bilateral absence of SVC in the literature.

Author	Years	Age	Gender	Symptoms	Medical history	Diagnostic exam
Hussain et al.^2^	1981	82 years	F	Syncopal attacks	Complete AV‐block with bradycardia	Venography
Del Ojo et al.^10^	1999	81 years	M	Dizziness	–	Venography
Saunders et al.^11^	2001	25 years	F	Dyspnea	Smoker	CT, MRI, venography, sternotomy
Minniti et al.^6^	2002	N/A	N/A	N/A	N/A	CT, venography
Krasemann et al.^12^	2003	3 months	M	Clinical signs of TOF	–	Venography, cardiopulmonary bypass
Lee et al.^13^	2005	1 week	F	Respiratory distress, upper body edema, chylothorax	–	Echo, CT, venography
Akai et al.^14^	2006	28 years	M	Asymptomatic	–	CT, MRI
Römer et al.^7^	2006	Prenatally 24 weeks	M	Prenatal bilateral fetal hydrothorax, postnatal respiratory distress and chylothorax	Sibling w/ Adams‐Oliver Syndrome	PPUS, MRI
Ou et al.^15^	2007	14 months	M	Asymptomatic cardiac murmur	–	Echo, CT
Quraishi et al.^16^	2010	59 years	M	Palpitations	Wolff‐Parkinson‐White	Venography, CT
Bansal et al.^3^	2012	Prenatally 24 weeks	M	Asymptomatic	–	PPUS, CT
Ylänen et al.^1^	2012	Prenatally 20 weeks	M	Cystic fetal hygroma	–	PPUS, CT and MRI
Park et al.^17^	2016	27 years	M	Chronic cough	Smoker (1 year)	CT
Shah et al.^18^	2018	2 years	F	Cyanosis in TOF	–	Open surgery, CT
Derbel et al.^8^	2019	43 years	F	Asymptomatic	–	CT
Zhang et al.^19^	2019	22 years	M	Facial and neck swelling	–	Echo, CT, Venography
Takajo et al.^20^	2021	24 months	M	N/A	N/A	Echo, MRI

Abbreviations: CT, computed tomography; Echo, echocardiography; F, female; M, male; MRI, magnetic resonance imaging; PPUS, pre‐ and post‐natal ultrasound; TOF, tetralogy of Fallot.

In cases of persistent SVC with an absent right SVC,^4^ younger patients with a bilateral absence of SVC tend to have structural pathologies, such as tetralogy of fallot.^12^, ^18^ Additionally in light of current literature, it is evident that generally congestive circulatory symptoms such as dyspnea, cyanosis, or upper body edema tend to appear in younger patients,^7^, ^12^, ^13^, ^18^, ^19^ whereas adult or older patients present with symptoms related to rhythm disturbances^2^, ^16^ or no related symptoms at all^8^, ^11^, ^14^, ^17^ rather than congestive circulatory symptoms.

In our view, the most likely explanation for this phenomenon in younger patients lies within the veins in the immediate vicinity of the missing SVC not being dilated or varicose. As such, the full blood volume of the upper limbs and head is forcibly drained through these veins at an ineffective rate, leading to congestion of blood proximally and thus to the congestive symptoms mentioned above. Dilation or varicosing of the aforementioned veins, in turn, arises as patients age as a compensatory mechanism of the body to accommodate this excess volume and avoid congestion and its associated symptoms, which was also seen in our patient.

Especially when faced with congestive circulatory symptoms, knowledge of the embryonic development is an important landmark when establishing a diagnosis/differential diagnosis cascade. The most cited differential diagnosis is SVC obstruction,^1^, ^17^ for example, due to infections, malignancies, heart surgery, or thrombosis. Therefore, asking the patient about specific signs and symptoms such as respiratory symptoms, upper body edema, cyanosis, or a positive Pemberton sign, as well as relevant prior medical, and surgical history is crucial for diagnostic purposes.

In light of current literature, suspicion either on chest x‐ray or ultrasound should be supplemented by CT angiography instead of angiography as previously recommended,^12^ mostly due to better technology in modern CT scanners with the possibility of reconstruction in thin slices, such as 1 mm or even 0.5 mm slice thickness without excess noise or loss of resolution. MRI is also a good radiation‐free alternative with possibility of 3D visualization, especially for younger patients. However, in addition to CT scans being faster^1^ and less claustrophobic than MRI scans, patient cooperation especially in breath‐adjusted sequences is a major differentiator, thus making diagnosis via MRI more exhausting.

We believe that this follow‐up diagnostic cascade gains greater importance in the field of pacemaker implantation, where increased resistance during lead implantation may lead to vascular wall lesion, as well as in the field of abdominal surgery in the preoperative setting, as certain procedures that require clamping of the IVC—for example, hepatectomy or radical nephrectomy—may lead to an increased engorgement of the draining veins, including the abnormally draining varicose collateral veins. In this case, since these veins have no rigid wall, an acute increase in blood pressure may eventually lead to spontaneous ruptures of these collaterals, which could otherwise be avoided in the knowledge of this circulatory variant's vascular topography.

Finally, CT angiography or MRI do not substitute the need to evaluate the patient with ultrasound, specifically echocardiography, due to the known association of SVC anomalies with cardiac or rhythm abnormalities. A causative pathophysiological mechanism associated with other abnormalities is, in light of current literature, not yet known. However, due to the association with cardiac and rhythm abnormalities, referral to a cardiologist or pediatric cardiologist is of great importance in the evaluation of patients of any age.

## 4 CONCLUSION

A bilateral absence of the SVC, albeit being an extremely rare congenital abnormality, yields a great importance when faced with difficult cardiac or venous procedures. Upon suspicion, patients' clinical, medical, and surgical histories are key diagnostic orientators and need to be assessed carefully. Full visualization of the venous anomaly, ideally through a CT scan, for future procedural and operative planning, as well as referral to a cardiologist for the determination/exclusion of a cardiac structural and rhythm abnormality are crucial in this setting.

## AUTHOR CONTRIBUTIONS

**Yosef Al‐Sewaidi:** Conceptualization; investigation; methodology; project administration; resources; writing – original draft; writing – review and editing. **Robert Smaczny:** Conceptualization; investigation; supervision; validation; writing – review and editing. **Alexander Ranft:** Investigation; supervision; validation.

## FUNDING INFORMATION

No funding was required for this case report.

## CONFLICT OF INTEREST STATEMENT

None to declare.

## CONSENT

Written informed consent was obtained from the patient to publish this study according to the patient consent policy. The authors declare that the patient's confidentiality has been respected.

## Data Availability

The data that support the findings of this study such as the patient's computed tomography scan are available from the corresponding author upon reasonable request.
